# Supplementary material for: Mechanisms governing the pioneering and redistribution capabilities of the non-classical pioneer PU.1
Source: Nat Commun. 2020 Jan 21;11:402. doi: 10.1038/s41467-019-13960-2 (PMC6972792; doi:10.1038/s41467-019-13960-2)
Supplement: Supplementary file 7 — Source data [file 41467_2019_13960_MOESM7_ESM.zip › Source_Data/FigureS2/FigureS2B_MotifScanOutput/KmeansCluster12/homerResults/motif4.similar.html]

motif4

## Information for motif4

A
C
T
G
A
G
T
C
A
G
C
T
A
G
C
T
A
G
T
C
A
G
T
C
C
A
G
T
A
G
T
C
G
T
A
C
G
A
T
C
A
T
G
C
G
A
T
C
  
Reverse Opposite:  

C
T
A
G
T
A
C
G
C
T
A
G
C
A
T
G
T
C
A
G
G
T
C
A
T
C
A
G
T
C
A
G
T
C
G
A
T
C
G
A
T
C
A
G
T
G
A
C
  

|  |  |
| --- | --- |
| p-value: | 1e-57 |
| log p-value: | -1.323e+02 |
| Information Content per bp: | 1.698 |
| Number of Target Sequences with motif | 388.0 |
| Percentage of Target Sequences with motif | 21.59% |
| Number of Background Sequences with motif | 4042.7 |
| Percentage of Background Sequences with motif | 8.79% |
| Average Position of motif in Targets | 99.2 +/- 53.7bp |
| Average Position of motif in Background | 100.3 +/- 70.1bp |
| Strand Bias (log2 ratio + to - strand density) | -0.3 |
| Multiplicity (# of sites on avg that occur together) | 1.10 |
| Motif File: | file (matrix) reverse opposite |

### Similar de novo motifs found

|  |  |  |  |  |  |  |  |
| --- | --- | --- | --- | --- | --- | --- | --- |
| Rank | Match Score | Redundant Motif | P-value | log P-value | % of Targets | % of Background | Motif file |
| 1 | 0.763 | A C T G A C T G A T C G A T C G A C T G C T A G C T A G C T A G T C G A | 1e-22 | -52.649153 | 27.88% | 18.08% | motif file (matrix) |
| 2 | 0.763 | T A C G A G C T A G C T A C G T A G T C A T G C G C A T A G T C A T G C A G T C A G C T C T A G | 1e-12 | -28.167637 | 2.23% | 0.52% | motif file (matrix) |
